# Histone-, Receptor-, and Integrin-Related Gene Products and ADAM28 as Relevant to B-Cell Acute Lymphoblastic Leukemia (B-ALL)

**DOI:** 10.3390/cimb47090699

**Published:** 2025-08-28

**Authors:** Makayla R. K. Wilkins, Brett E. Pickett

**Affiliations:** Department of Microbiology and Molecular Biology, Brigham Young University, Provo, UT 84062, USA

**Keywords:** B-ALL, mechanisms, bulk RNA-sequencing, signaling pathways, therapeutics, diagnostics

## Abstract

Acute lymphoblastic leukemia (ALL) is the most common childhood cancer, with pediatric ALL having a ~90 percent cure rate, while the adult cure rate is considerably lower. B-cell acute lymphoblastic leukemia (B-ALL) is the most common subtype of ALL and is generally treated through a variety of chemotherapy drugs that can cause undesired side effects, adverse events, or other complications. Consequently, there is a need for improved understanding of the shared gene expression profiles and underlying molecular mechanisms shared among various B-ALL subtypes. In this study, 259 publicly available RNA-sequencing samples were evaluated and retrieved from the NCBI Gene Expression Omnibus (GEO) database and then pre-processed using a robust computational workflow. Differential gene expression, pathway enrichment, marker prediction, and drug repurposing analyses were then performed to facilitate a better mechanistic understanding of disease. We found both previously identified as well as novel differentially expressed genes. Specifically, we observed upregulation in the *HIST2H2AA3*, *EPHA7*, and *MPR1* genes; while downregulation was observed for the *IGHA1*, *ANGPTL1*, and *CHAD* genes. We identified multiple pathways, including “Integrins in Angiogenesis”, to be significantly affected in B-ALL. We then used these significant pathways to predict and rank 306 existing therapeutic targets that could potentially be repurposed for B-ALL, including three that have not been evaluated in human clinical trials. Using a tree-based classification algorithm, we also predicted ADAM28 as a possible mechanistic marker. The results of this study have potential implications for patients who have been diagnosed with B-ALL by providing improved mechanistic understanding and information on possible diagnostics and repurposed therapeutics for B-ALL.

## 1. Introduction

Acute lymphoblastic leukemia (ALL) is the most common childhood cancer, with B-cell acute lymphoblastic leukemia (B-ALL) being the most common subtype [1,2]. B-ALL, which occurs in B-cell progenitor cells, is responsible for about 75% of adult ALL diagnoses [3]. In pediatric ALL cases, the cure rate is ~90% [2], while the cure rate in adult ALL is ~30–50% [3,4].

Although the survival rate of acute lymphoblastic leukemia has improved in recent years, the standard therapy, which includes a combination of various chemotherapy agents, such as vincristine, anthracycline, L-asparaginase, and methotrexate, can lead to a variety of negative side-effects, including both short-term and long-term complications [1,3,4,5,6,7,8,9]. Thus, a need still exists to identify improved treatments that can increase the quality of life for B-ALL patients both during and after treatment and increase the cure rate, especially for adults. Such advances in the discovery and development of new therapeutic options can be achieved through increased understanding of the underlying pathogenic mechanisms of B-ALL. Given the relatively small number of human studies involving primary cells, performing a secondary analysis of multiple public datasets is justified.

The purpose of the current study was to perform a large-scale comparison of the gene expression patterns between B-cells from patients with B-ALL vs. B-cells from healthy patients to better understand the common underlying molecular mechanisms of disease, as well as to identify any shared transcriptional diagnostic markers in a large set of B-ALL patients regardless of molecular type. We report our findings of the significant differentially expressed genes (DEGs) found in B-ALL, as well as significant signaling pathways, potential targets for drug repurposing, and one mechanistic marker derived from the compared transcriptomic data. We believe these findings will be useful in the future development of therapeutic targets that can increase efficiency in treating B-ALL.

## 2. Materials and Methods

### 2.1. Data Acquisition

Samples were gathered from publicly available data found from the National Center for Biotechnology Information (NCBI) Gene Expression Omnibus (GEO) database [10]. Specifically, we identified relevant studies and samples by applying search criteria including “Homo sapiens” and “gene expression using high-throughput sequencing” and manual selection to rule out cell lines, patient-derived xenograft samples, treated cells, relapsed samples, and single-cell RNA-sequencing data to obtain the desired RNA-sequencing reads for B-ALL and non-diseased B-cell samples. The fastq files for these RNA sequencing data were downloaded from the Sequence Read Archive (SRA) database [11]. The B-ALL samples were obtained from three datasets (GSE156531 [12], GSE124824 [13], and GSE79373 [14]), which labeled samples as “B cell acute lymphoblastic leukemia,” “B-lineage ALL,” and “precursor B-cell acute lymphoblastic leukemia” respectively, and the healthy B-cell samples were likewise acquired from three different datasets (GSE115655 [15], GSE181859 [16], and GSE149050 [17]). All B-ALL samples were collected from the bone marrow with 25 known pediatric samples, one sample from a 19 year-old patient, and 197 samples of unknown age. These 197 samples came from a larger dataset mixed with pediatric and adult data; however, the latter dataset lacked age-related metadata. Thus, the exact counts in each age category are unknown. Of the 36 controls, four came from bone marrow samples and 32 came from peripheral blood samples. There were two known pediatric samples, 14 adult samples, and 20 of unknown age.

### 2.2. Preprocessing of RNA-Sequencing Data

These fastq files were run through the existing Automated Reproducible MOdular workflow for preprocessing and differential analysis of RNA-seq data (ARMOR) v1.5.10 bioinformatics workflow [18]. The ARMOR pipeline performs quality control on the RNA-seq reads using FastQC v0.11.9, trims adapters and poor-quality regions of the reads with TrimGalore! v0.6.6, quantifies and maps reads to the human transcriptome with Salmon v1.4.0 [19], calculates differential expression using edgeR v4.2.2 [20], and computes significantly enriched Gene Ontology terms with Camera v3.60.6 [21]. A metadata file and configuration file were created to provide appropriate instructions for this workflow and assign fastq files to their respective groups, B-ALL or control.

### 2.3. Downstream Analyses

The differentially expressed genes calculated using ARMOR were then analyzed using the Signaling Pathway Impact Analysis v2.54.0 (SPIA) program to generate a null distribution for each pathway based on the input genes through permutation and bootstrapping [22]. The signaling pathways were retrieved from five public databases, including KEGG, Reactome, and NCI, as has been done previously [23,24]. The significant signaling pathway results that were generated with SPIA were then used as input for the Pathway2Targets algorithm [25,26]. Briefly, this algorithm uses a customizable weighting scheme to rank potential therapeutics by various attributes in the Open Targets database that could be repurposed to target members of the significantly altered B-ALL pathways. Lastly, the Salmon gene counts data from the ARMOR pipeline was used as the input for the tree-based machine learning workflow to predict transcriptional mechanistic markers of B-ALL disease.

## 3. Results

### 3.1. Unsupervised Clustering Reveals Separation of Case and Control Samples

**Figure 1:**
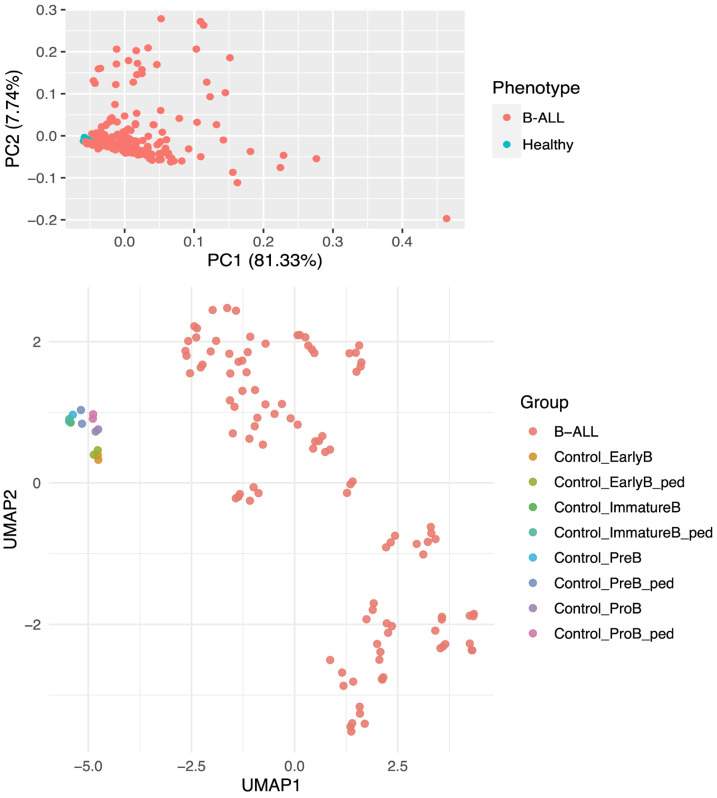
Dimensional reduction analyses of all samples. (Upper Panel) Principal component analysis (PCA) of all samples. B-ALL samples (red) and healthy control samples (blue) show the variance-based differences between the phenotypes. (Lower Panel) UMAP plot with control samples clustering towards the *Y*-axis and B-ALL samples being more diverse and further from the *Y*-axis. Phenotypes for control cells include early B-cells (Control_EarlyB), pediatric early B-cells (Control_EarlyB_ped), immature B-cells (Control_ImmatureB), pediatric immature B-cells (Control_ImmatureB_ped), pre-B cells (Control_PreB), pediatric pre-B cells (Control_PreB_ped), pro-B cells (Control_ProB), and pediatric Pro-B cells (Control_ProB_ped).

To determine whether the samples in this study formed separate clusters based on disease state, we began by performing a principal component analysis of all included samples. These PCA results showed the potential for two clusters based on the phenotype (Figure 1). We calculated that 89.07% of the variance between samples was explained by the first two principal components. Specifically, principal component one explained 81.33% of the variance, while principal component two explained an additional 7.74% of the variance. We next generated a Uniform Manifold Approximation and Projection (UMAP) plot in an attempt to improve the resolution. The UMAP shows that the control samples clustered relatively closely to each other, while the B-ALL samples were much more diverse. Although this observation was partially expected, the diversity of the B-ALL samples could be due to differences in one or more factors, including molecular subtype, genetic aberrations, and/or cancer stage. These results suggest sufficient transcriptional differences to justify downstream analyses of the transcriptional profiles comparing B-ALL and healthy B-cells.

### 3.2. Differential Gene Expression Highlights Significant Differences in B-ALL

**Figure 2:**
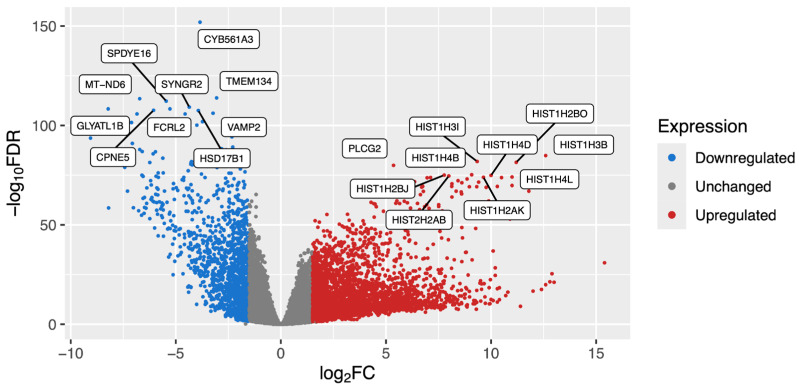
Volcano plot of B-ALL DEGs. Labels are included for the top 20 most statistically significant protein-coding genes that were either upregulated (red) or downregulated (blue). Higher values on the *Y*-axis represent more significant *p*-values, while the *X*-axis represents positive (**right**) or negative (**left**) log_2_ fold-change values.

To elucidate the transcriptional differences between B-ALL and healthy B-cells, we applied a robust computational workflow to process and analyze RNA-sequencing data collected from publicly available clinical samples. Our analysis, which consisted of 223 B-ALL samples and 36 healthy B-cell samples, revealed over 8000 statistically significant differentially expressed genes (FDR-adjusted *p*-value < 0.05), and a log_2_ fold change (log_2_FC) value greater than the absolute value of one (Figure 2, Supplementary Materials). Unexpectedly, approximately 1000 of these statistically significant DEGs were pseudogenes.

We found that the most statistically significant six protein-coding DEGs that were upregulated in B-ALL samples included two histone gene products named histone cluster 2 H2A family member A3 and istone Cluster 1 H3 family member B (*HIST2H2AA3* and *HIST1H3B* respectively); C-type lectin domain containing 14A (*CLEC14A*), which is a gene product involved in endothelium and angiogenesis; as well as other gene products that contribute receptor binding such as ephrin receptor A7 and natriuretic peptide receptor 1 (*EPHA7*, *NPR1* respectively) (Table 1).

The top six statistically significant protein-coding DEGs found to be downregulated in B-ALL samples were angiopoietin-like 1 (*ANGPTL1*), as well as gene products that function in immunoglobulin chain formation-associated immunoglobulin heavy constant alpha 1 and immunoglobulin kappa variable 3D-11 (*IGHA1* and *IGKV3D-11*, respectively), adhesion-associated chondroadherin (*CHAD*), glycine-N-acyltransferase-like 1B (*GLYATL1B*), and a churchill domain containing 1-farnesyltransferase fusion gene product (*CHURC1-FNTB*) (Table 2).

### 3.3. Signaling Pathway Enrichment Identifies Eight Biologically Relevant Cascades

We next used the SPIA algorithm, which uses protein–protein interactions and topology-based information to identify intracellular signaling pathways that are significantly enriched in our DEGs. Briefly, this algorithm uses permutation and bootstrapping to generate a null statistical distribution for each pathway. Our pathway analysis identified eight significantly enriched pathways that were significantly enriched in our list of DEGs. Such pathways included the “integrins in angiogenesis” pathway (false discovery rate-adjusted *p*-value 8.565 × 10^−3^), “Antigen processing and presentation” (FDR-adjusted *p*-value 7.797 × 10^−4^), “Calcium signaling pathway” (FDR-adjusted *p*-value 3.862 × 10^−3^), and others (Table 3).

### 3.4. Therapeutic Target Prioritization Predicts Known and Novel Targets

We next wanted to determine whether the signaling pathway results could be used to predict therapeutic targets that could be repurposed for B-ALL. Although the “integrins in angiogenesis” pathway did not contain any existing targets, we obtained 306 total therapeutic targets using the Pathways2Targets algorithm. A subset of the highest-ranked predicted targets for repurposing include tumor necrosis factor-alpha (TNF-alpha), interleukin-6 (IL-6), tumor protein p53 (TP53), and AKT serine/threonine kinase 1 (AKT1); which could potentially be repurposed as protein targets for B-ALL (Table 4).

We also identified FMS-related receptor tyrosine kinase 1 (FLT1) as a potential target for repurposing, which previously has not been evaluated for B-ALL in clinical trials. Our effort to identify more early-stage targets for repurposing consisted of filtering the Pathway2Targets results to remove those that have been (1) FDA approved for at least one indication, (2) evaluated in a phase three clinical trial, and (3) involved in at least five phase two clinical trials. This approach produced a subset of predicted targets for repurposing that included cluster of differentiation-40 (CD40), toll-like receptor 8 (TLR8), and glutamate metabotropic receptor 5 (GRM5). None of these three targets have been evaluated in clinical trials for B-ALL previously.

### 3.5. Machine Learning Identified ADAM28 as a Mechanistic Marker

To determine potential mechanistic markers that display more consistent patterns across B-ALL and healthy controls, we used the RNA-sequencing gene counts data as input to a tree-based machine learning analysis implemented in the xgboost R library to detect additional gene products that play a mechanistic role in B-ALL. This analysis identified ADAM metallopeptidase domain 28 (ADAM28) as a putative mechanistic marker. Our analysis showed this potential marker to have both a specificity and sensitivity of 1.0, meaning that this one gene product could potentially predict with 100% accuracy whether a patient in our multi-study dataset had B-ALL. We found this gene to be significantly downregulated in our B-ALL analysis, with a log_2_FC of −6.08 (FDR-adjusted *p*-value 1.92 × 10^−86^). This gene product was not in the most downregulated DEGs reported above, which was expected given that this approach applied machine learning to identify the gene product that was most consistently found in the disease phenotype rather than those that significantly differed between the case and control groups.

## 4. Discussion

The goal of this work was to perform a secondary analysis of 259 existing publicly available RNA-sequencing samples comparing patients with B-ALL to healthy controls. The novelty of our approach includes multiple samples across various studies, which potentially increases the statistical power across a larger population of humans. As such, our findings include both previously reported DEGs, as well as novel DEGs that may contribute to the characteristics shared between the various B-ALL types. We identified multiple significant intracellular mechanistic signaling pathways, as well as 306 therapeutic targets that could be relevant to B-ALL. Among this list of existing therapeutic targets are FLT1, CD40, TLR8, and GRM5, which could potentially be repurposed specifically for B-ALL. We also report the results from a tree-based machine learning exercise that identified the ADAM28 gene product as a potential mechanistic marker, which was downregulated in B-ALL.

*HIST2H2AA3* was the most upregulated gene in our differential expression analysis. Although there is currently a lack of direct connections between *HIST2H2AA3* and B-ALL, there is evidence that this gene is altered in peripheral blood mononuclear cells after exposure to benzene, which has been linked to leukemia [27], and shows a potentially noteworthy mechanism to explore in future experiments.

The ephrin type-A receptor 7 (*EPHA7*) gene was also highly upregulated in our differential expression analysis. This gene product is part of the Eph/Ephrin class of receptors and genes, which are tyrosine kinase receptors, and contribute to development and growth [28,29]. The EPHA7 gene product has been previously found to be upregulated in B-ALL in a comparison between pediatric patients with B-ALL and pediatric patients with other common blood conditions [30]. Another study found *EPHA7* to be upregulated in B-ALL (compared to healthy samples), which is consistent with our findings [29]. It should be noted that a prior study found *EPHA7* to be methylated in several leukemia cell lines and acute lymphoblastic leukemia patient samples [28], which may be dependent on various experimental conditions.

The natriuretic peptide receptor 1 (*NPR1*) gene was third-highest on our list of differentially expressed genes. This gene product is a membrane-bound receptor for the vasoactive hormones atrial natriuretic peptide (ANP) and brain natriuretic peptide (BNP) and is a guanylyl cyclase that has previously been associated with B-ALL [31,32]. Interestingly, prior work observed that *NPR1* was upregulated in B-lineage ALL, with no added chromosomal abnormalities compared to three other main subtypes of B-lineage ALL [33], while a separate study found *NPR1* to be upregulated in mixed-lineage leukemia gene rearrangement (MLL-R) ALL [34]. Our analysis, which focused specifically on B-cell ALL, found *NPR1* to be upregulated in B-ALL when compared to healthy controls, which is novel. Additional work is needed to better characterize the role of *NPR1* in B-ALL.

Divergent protein kinase domain 1C (*DIPK1C*) was also identified as upregulated in our differential expression analysis. This gene product is found in the endoplasmic reticulum, though its function has not been fully characterized. One prior study found elevated expression of *DIPK1C* in precursor B-ALL (BCP-ALL) samples that were sensitive to a second mitochondria-derived activator of caspase mimetic therapy [35]. Further evaluation of the mechanisms of this gene in B-ALL could prove fruitful in developing targeted cancer therapies for B-ALL cases.

We also identified histone cluster 1 H3 family member B (*HIST1H3B*) to be strongly upregulated in the B-ALL vs. healthy control comparison. This gene was previously found to be a DEG in both adult and pediatric acute myeloid leukemia (AML) [36]; however, overexpression of this gene product in B-ALL is novel. *CLEC14A* was also highly upregulated, and its gene product is found on the surface of endothelial cells in the blood vessels. It was found to be overexpressed in the vasculature of various solid tumors [37], as well as in two subtypes of precursor B-ALL in samples with high expression of binder of MAP3K1 and KLF4 (*BAALC*) [38]. However, we did not find previous literature that compared *CLEC14A* expression in B-ALL to healthy samples, as was done in the current work.

The *ANGPTL1* gene was found to be highly downregulated in our analysis. Its gene product is part of the angiopoietin family, which contains factors involved in vascular growth. This gene has been shown to inhibit solid tumor metastasis and has been shown to be relevant in other cancers [39,40], although relatively little is known about its role in B-ALL [39,40,41,42,43]. One prior study found this gene product to be upregulated in multiple cancer types, including AML, chronic lymphocytic leukemia (CLL), T-cell lymphoma, unspecified ALL, pre B-ALL, and T-cell ALL. Additional experiments in the wet lab will help to better elucidate the role of this gene product in B-ALL.

*CHAD* was also highly downregulated in our analysis. The gene product has been shown to bind to chondrocytes through the α2β1 integrin [44], while GLYATL1B is thought to be involved in activities related to glutamine processing. Our observation that *CHAD* and *GLYATL1B* are significantly downregulated in B-ALL is novel.

*CHURC1-FNTB* was also on our list of downregulated genes. This readthrough results from a fusion gene consisting of the churchill domain containing 1 gene and the farnesyltransferase, CAAX box, subunit beta gene. The first of these two gene products contributes to regulation of transcription and mediating fibroblast growth factor (FGF) signaling and binds zinc [45]. The second contributes to protein farnesylation and also binds zinc ions for function [46]. Our finding that *CHURC1-FNTB* is differentially expressed in B-ALL is novel, though work has been done to pursue this target as a cancer immunotherapy for a variety of different cancers, including AML [47,48].

We also found *IGHA1* and *IGKV3D-11* to be downregulated in B-ALL. A prior study in chronic lymphocytic leukemia found *IGKV3D-11* to be downregulated [49]. In addition, prior proteomic analysis of human MLL-R BCP-ALL samples vs. healthy precursor B-cell samples, found *IGHA1* to be downregulated [50]. However, though we believe this to be a novel finding in B-ALL, the abnormal expression of immunoglobulins in cancerous B-cells is not surprising.

Our pathway enrichment analysis identified eight intracellular signaling pathways that were dysregulated in B-ALL, including “Integrins in angiogenesis”. There have been many prior studies that have associated angiogenesis or integrins to the progression of B-ALL [51,52]. Interestingly, a previous study that treated B-ALL cell lines with an anti-alpha-6 integrin antibody observed a significant decrease in cell survival [53]. Overall, this demonstrates that this pathway is one area of further research that could improve the effectiveness of B-ALL treatment when included with conventional drugs, even though it contains no existing therapeutic targets. A subset of the remaining significantly affected pathways such as “Systemic lupus erythematosus” and “Epstein-Barr virus infection” have previously been associated with B-ALL [54,55,56,57], while pathways such as the “NF-kappa B signaling pathway” and “Toll-like receptor signaling pathway” are known to play a role in innate immunity and have been associated with B-ALL in prior studies [58,59,60,61]. Additional experiments are needed to better characterize the role of these pathways in B-ALL.

Some of the established therapeutic targets that were highly ranked in our B-ALL target repurposing analysis include TNF-alpha [62,63,64], IL-6 [65,66], TP53 [67,68], AKT1 [69], and BCL2 [70]. Interestingly, FLT1 was also predicted to be a relevant target for B-ALL. FLT1 is a cell-surface receptor for vascular endothelial growth factors (VEGFs) A and B, which play roles in cell survival and migration as well as chemotaxis and cancer cell invasion. FLT1 is also a receptor for placental growth factor (PGF), which positively regulates cell proliferation and tumor growth. FLT1 has been evaluated as a target in other cancers (e.g., acute myeloid leukemia, leukemia, and chronic lymphocytic leukemia) and has been reported to be an important gene product in B-ALL [30,33,71,72]. However, using this target as a potential target for B-ALL has not been evaluated in human clinical trials (platform.opentargets.org).

Identifying CD40, TLR8, and GRM5 from the list of filtered therapeutic targets that have been tested in multiple phase-two trials is both interesting and biologically relevant. CD40, which is a member of the tumor necrosis factor receptor superfamily, transduces ERK-activating signals in B cells to induce secretion of immunoglobulins. This therapeutic target has been evaluated in a phase-two clinical trial for diffuse large B-cell lymphoma, and another trial for Sjogren syndrome. It has also been involved in phase one clinical trials for chronic lymphocytic leukemia, but for no other subtype of leukemia, including B-ALL. Interestingly, CD40 has been reported as playing a role in B-ALL [59,73], with recent work in a patient-derived xenograft mouse model showing that a CD40 agonist could effectively target B-ALL cells [74].

TLR8 is known to play a key role in the innate immune response, with evidence of agonists contributing to progression of B-ALL [75], and the potential to enable lymphocytes to infiltrate solid tumors [76]. GRM5 is a G-protein-coupled receptor for glutamate, which is generally associated with neurons. Binding of this receptor activates the phosphatidylinositol system to generate a calcium-activated chloride current. This gene product has previously been associated with B-ALL [77], though its role has not been well characterized. While the results from our target repurposing analysis at least partially reflects the underrepresentation of all gene products across existing curated pathways, we believe that the high-ranked targets from this analysis particularly warrant validation in subsequent wet-lab experiments.

Lastly, the *ADAM28* gene, which was identified by our machine learning approach to predict mechanistic markers, has been shown in various cancers [78,79,80]. Past research on this gene product in solid (lung) tumors showed that it may act as a tumor suppressor [81], and that ADAM28 protein levels were higher in adjacent healthy tissues in human colorectal cancer tumors [82]. ADAM28 has also been reported as having a role in inducing leukemic cell proliferation in B-AML [83]. Prior work has shown that bone marrow levels of ADAM28 were increased in recently diagnosed B-ALL patients when compared to healthy controls, and that ADAM28 expression was significantly higher in B-ALL patients who relapsed compared to patients who continued in remission [84]. However, it was unclear if the increased ADAM28 expression in the latter study was specifically due to B cells, which could be one potential factor that contributed to our differing results. Overall, this and other studies support ADAM28 as a potential mechanistic marker for B-ALL. Nevertheless, additional characterization experiments are required to determine whether ADAM28 could have diagnostic and/or prognostic potential in at least a subset of pediatric B-ALL cases.

There are potential limitations to this study, which primarily revolve around the use of publicly available bulk RNA-sequencing data with inconsistent metadata. One such limitation is that the ethnicity and sex of the patients from whom the samples were collected were unknown in the majority of the datasets, as well as the patient age in a subset of the datasets. In addition, only bone marrow B-ALL samples were used, but the controls contained a mixture of bone marrow and peripheral blood-derived B-cells. Even given these potential limitations, our results from 259 samples replicate findings from past work and include novel results. This suggests that the findings from this study are likely to be relevant to B-ALL, although additional validation experiments in multiple patient cohorts are still needed to validate the findings of this study.

## 5. Conclusions

Overall, this study identified statistically significant differentially expressed genes between B cells from patients with acute lymphoblastic leukemia and healthy B cells. The top DEGs analyzed included four genes with differential expression that have been previously associated with B-ALL, which shows the validity of our approach. In addition, eight of the DEGs in our B-ALL cells vs. normal B-cells are novel in B-ALL and could be investigated further in the context of B-ALL. We also found the “integrins in angiogenesis” and other pathways to be significantly dysregulated during this disease. Our results have predicted several potential therapeutic targets that could be repurposed for B-ALL, and that the ADAM28 gene product could potentially predict B-ALL with 100 percent accuracy in our combined dataset. These results suggest a continued need for continued research into the underlying mechanisms for B-ALL in bone marrow and could potentially be applied to the development and/or repurposing of targets to improve B-ALL treatment in future experiments.

## Figures and Tables

**Table 1:** Top 10 upregulated protein-coding DEGs together with false discovery rate-adjusted *p*-value (FDR *p*-value) calculated in the B-ALL vs. healthy control comparison, sorted in decreasing order by positive log_2_ fold-change values.

Gene Symbol	Gene Name	log2FC ^1^	FDR ^2^
*HIST2H2AA3*	histone cluster 2 H2A family member a3	15.4	1.20 × 10^−31^
*EPHA7*	EPH receptor A7	13	7.63 × 10^−22^
*NPR1*	natriuretic peptide receptor 1	12.9	3.69 × 10^−26^
*DIPK1C*	divergent protein kinase domain 1C	12.8	4.31 × 10^−22^
*HIST1H3B*	histone cluster 1 H3 family member b	12.6	1.49 × 10^−85^
*CLEC14A*	C-type lectin domain containing 14A	12.6	1.28 × 10^−20^
*S100A16*	S100 calcium binding protein A16	12.4	3.06 × 10^−18^
*OVCH2*	ovochymase 2 (gene/pseudogene)	12	2.14 × 10^−17^
*ERG*	ETS transcription factor ERG	11.8	1.16 × 100^−67^
*GREM1*	gremlin 1, DAN family BMP antagonist	11.4	9.29 × 100^−10^

^1^ log_2_ fold change. ^2^ false discovery rate-corrected *p*-value.

**Table 2:** Top 10 downregulated protein-coding DEGs together with false discovery rate-adjusted *p*-value (FDR *p*-value) calculated in the B-ALL vs. healthy control comparison, sorted in increasing order by negative log_2_ fold-change values.

Gene Symbol	Gene Name	log2FC ^1^	FDR ^2^
*IGHA1*	immunoglobulin heavy constant alpha 1	−9.56	9.11 × 10^−91^
*ANGPTL1*	angiopoietin like 1	−9.06	2.38 × 10^−94^
*IGKV1-39*	immunoglobulin kappa variable 1–39 (gene/pseudogene)	−8.48	2.43 × 10^−65^
*GLYATL1B*	glycine-N-acyltransferase like 1B	−8.22	4.93 × 10^−109^
*CHAD*	chondroadherin	−8.22	1.48 × 10^−87^
*CHURC1-FNTB*	CHURC1-FNTB readthrough	−8.2	3.11 × 10^−59^
*IGKV3D-11*	immunoglobulin kappa variable 3D-11	−8.08	3.67 × 10^−75^
*AC011511.4*	novel transcript	−7.94	6.39 × 10^−72^
*IGKV2D-28*	immunoglobulin kappa variable 2D-28	−7.87	2.33 × 10^−73^
*ABHD16B*	abhydrolase domain containing 16B	−7.73	1.25 × 10^−102^

^1^ log_2_ fold change. ^2^ false discovery rate-corrected *p*-value.

**Table 3:** Intracellular signaling pathways that were significantly enriched in DEGs.

Name	Number of Proteins in Pathway	Number of DEGs in Pathway	FDR *p*-Value	Pathway Regulation Status	Source Database
Integrins in angiogenesis	54	40	8.565 × 10^−3^	Activated	NCI
Antigen processing and presentation	63	55	7.797 × 10^−4^	Inhibited	KEGG
Calcium signaling pathway	134	102	3.862 × 10^−3^	Activated	KEGG
Systemic lupus erythematosus	119	109	2.521 × 10^−2^	Activated	KEGG
NF-kappa B signaling pathway	85	76	4.272 × 10^−2^	Inhibited	KEGG
Toll-like receptor signaling pathway	85	76	4.354 × 10^−2^	Inhibited	KEGG
Tight junction	105	97	4.354 × 10^−2^	Activated	KEGG
Epstein–Barr virus infection	195	159	4.449 × 10^−2^	Inhibited	KEGG

**Table 4:** Highest-ranked 10 therapeutic target proteins to potentially be repurposed for B-ALL.

Target Symbol	Target Name	Subcellular Location	Weighted Score
TNF	tumor necrosis factor	Cell membrane	3870
IL6	interleukin 6	Secreted	3287.5
TP53	tumor protein p53	Cytoplasm	3177
EGFR	epidermal growth factor receptor	Cell membrane	2562
CD40LG	CD40 ligand	Cell membrane	2490
AKT1	AKT serine/threonine kinase 1	Cytoplasm	2323.5
PIK3CA	phosphatidylinositol-4,5-bisphosphate 3-kinase catalytic subunit alpha	Cytosol	2301
PTGS2	prostaglandin-endoperoxide synthase 2	Microsome membrane	2288
PIK3CB	phosphatidylinositol-4,5-bisphosphate 3-kinase catalytic subunit beta	Cytoplasm	2228
PIK3CD	phosphatidylinositol-4,5-bisphosphate 3-kinase catalytic subunit delta	Cytoplasm	2142

## Data Availability

The raw data supporting our reported results can be found in the NCBI Gene Expression Omnibus (GEO) database using the following identifiers: GSE156531 [12], GSE124824, and GSE79373,GSE115655, GSE181859, and GSE149050.

## Supplementary Materials

The following supporting information can be downloaded at: https://www.mdpi.com/article/10.3390/cimb47090699/s1.

## Abbreviations

The following abbreviations are used in this manuscript:

B-ALL	B-cell acute lymphoblastic leukemia
GEO	Gene Expression Omnibus
DEG	Differentially expressed genes
SRA	Sequence read archive
ARMOR	Automated Reproducible Modular workflow for preprocessing and differential analysis of RNA-seq data
SPIA	Signaling pathway impact analysis
PCA	Principal component analysis
UMAP	Uniform manifold approximation and projection
FDR	false discovery rate-adjusted p-value
HIST2H2AA3	Histone cluster 2 H2A family member A3
HIST1H3B	H3 clustered histone 2
CLEC14A	C-type lectin domain containing 14A
EPHA7	Ephrin receptor A7
NPR1	natriuretic peptide receptor 1
ANGPTL1	Angiopoietin-like 1
IGHA1	Immunoglobulin chain formation-associated immunoglobulin heavy constant alpha 1
IGKV3D-11	Immunoglobulin kappa variable 3D-11
CHAD	Adhesion-associated chondroadherin
GLYATL1B	Glycine-N-acyltransferase like 1B
CHURC1-FNTB	Churchill domain containing 1-farnesyltransferase fusion
TNF	Tumor necrosis factor alpha
IL6	Interleukin 6
p53	Tumor protein p53
AKT1	AKT serine/threonine kinase 1
FLT1	FMS-related receptor tyrosine kinase 1
CD40	Cluster of differentiation 40
TLR8	Toll-like receptor 8
GRM5	Glutamate metabotropic receptor 5
ADAM28	ADAM metallopeptidase domain 28
DIPK1C	Divergent protein kinase domain 1C
BCP-ALL	Precursor B-ALL
BAALC	BAALC binder of MAP3K1 and KLF4
MLL-R	mixed-lineage leukemia gene rearrangement
AML	Acute myeloid leukemia
BCL2	BCL2 apoptosis regulator
